# Larval Pollen Stress Increases Adult Susceptibility to Clothianidin in Honey Bees

**DOI:** 10.3390/insects10010021

**Published:** 2019-01-08

**Authors:** Christina L. Mogren, Robert G. Danka, Kristen B. Healy

**Affiliations:** 1 Department of Entomology, Louisiana State University AgCenter, Baton Rouge, LA 70803, USA; khealy@agcenter.lsu.edu; 2 Plant and Environmental Protection Sciences, University of Hawaii at Mānoa, Honolulu, HI 96822, USA; 3 USDA-ARS, Honey Bee Breeding, Genetics, and Physiology Laboratory, Baton Rouge, LA 70820, USA; bob.danka@ars.usda.gov

**Keywords:** *Apis mellifera*, neonicotinoid, nutrition, sublethal effects, superoxide dismutase

## Abstract

Neonicotinoid insecticides have come under scrutiny for their potential role in honey bee declines. Additionally, reduced access to forage in agricultural areas creates the potential for risk interactions with these pesticides in regions critical for honey production. In this study, we sought to determine whether sufficient access to pollen during larval development could mitigate stress associated with oral clothianidin exposure in honey bee adults. An apiary was established where pollen traps deprived half of the colonies of pollen, which was then supplemented to the others. Adults were fed 0, 10, 40, 200, or 400 µg/L clothianidin in the laboratory, and larval and adult lipids and superoxide dismutase (SOD) activities were compared between feeding treatments. Survival at sublethal concentrations of clothianidin was significantly reduced for adult bees reared in pollen deprived colonies. Adult SOD activity was affected by clothianidin dose but not larval feeding treatment, though within the pollen-deprived cohort, SOD was greater in controls than those fed clothianidin. Larval SOD differed between field replicates, with supplemented colonies having slightly higher activity levels during a period of pollen dearth, indicating that supplementation during these periods is particularly important for mitigating oxidative stress within the hive. Larval lipids were significantly higher in supplemented colonies during a substantial pollen flow, though adult lipids were unaffected by feeding treatment. These results suggest that during periods of pollen dearth, oxidative stress and adult worker longevity will be improved by supplementing colonies with locally collected pollen.

## 1. Introduction

The interaction of nutrition and pesticide stress is of concern due to changing agricultural practices. Expanding monocultures of corn and soybeans in North America have led to reduced forage quality in the environment, particularly in the Northern Great Plains [1,2], which coincides with increased prophylactic use of neonicotinoid seed treatments in these major row crops [3]. The pesticides from these seed treatments do not stay in agricultural fields [4], and are found to contaminate pollen and nectar in non-target forage plants [5,6]. In honey bees (*Apis mellifera* L.), sublethal exposures to neonicotinoids have been demonstrated to affect reproduction, susceptibility to pathogens [7], locomotor abilities, and navigation [8]. In some cases, exposures have reduced colony-level survival and performance [9]. Given the value of these same agricultural areas in the Great Plains for honey production, resulting in high densities of honey bee colonies during the growing season [10], it is important to gauge the consequences resulting from neonicotinoid exposures in nutritionally stressed bees.

Access to abundant, high-quality pollen plays a critical role in honey bee health [11]: well-balanced diets are crucial for basic physiological functioning, development, and longevity of individual bees, as well as population growth and productivity of colonies. Honey bees consume pollen to obtain the vast majority of their nutritional requirements of protein, lipids, vitamins, and minerals (reviewed by Winston [12]). Additionally, micronutrient constituents in pollen may confer resilience towards various biotic and abiotic stressors, including pesticides [13].

The ability to cope with chemical toxins is an important protein-mediated stress response, and evidence suggests that well-nourished adult bees are less prone to damage from pesticides. For example, Wahl and Ulm [14] found that worker bees fed sufficient pollen as young adults were less sensitive to several insecticides, fungicides, and herbicides compared to less well-fed bees. Elsewhere, high protein diets have been shown to mitigate mortality associated with nicotine (when combined with cold temperature stress) and imidacloprid specifically [15,16]. Diets high in pollen have also resulted in upregulation of genes hypothesized to be involved in pesticide detoxification, thus reducing the sensitivity of worker bees to pesticides [17].

Numerous studies have evaluated the effects of neonicotinoid exposures on the locomotor abilities of honey bees [8,18,19]. Tosi, Burgio [20] found increased excitation in honey bees fed thiamethoxam at field-realistic concentrations. This in turn increases metabolic rates and thus the potential for the generation of reactive oxygen species, which damage lipids, proteins, and nucleic acids [21]. The increased production of superoxide dismutase (SOD) and other antioxidants by honey bees helps to neutralize these negative effects [22,23]. There is some evidence to suggest that SOD production varies in response to imidacloprid exposure and different diets, and could therefore be further developed as a biomarker to identify oxidative stress in response to pesticide exposures [16].

Despite a well-evidenced understanding of the effects of pollen feeding on adults and the resultant effects of pesticide susceptibility, to date there has been no work specifically investigating the role of larval pollen deprivation on adult insecticide susceptibility. In this study, we evaluate the prediction that colony-level pollen stress will result in weaker honey bees that are more susceptible to sublethal dietary clothianidin, a common neonicotinoid, as adults. Our objectives were two-fold: to determine whether adult mortality associated with environmentally relevant concentrations of clothianidin was higher in bees from nutritionally stressed colonies, and to evaluate lipid and superoxide dismutase levels in larvae and adults as sublethal markers to determine whether nutrition can be used to mitigate risks associated with clothianidin exposures.

## 2. Materials and Methods

### 2.1. Experimental Colonies

Twenty nucleus colonies were purchased from Merrimack Valley Apiaries in Bunkie, Louisiana, USA in March of 2017 with Italian (*A. mellifera ligustica*) and Carniolan (*A. mellifera carnica*) hybrid sister queens. Colonies were established and maintained at the USDA-ARS Honey Bee Breeding, Genetics, and Physiology Laboratory in Baton Rouge, Louisiana under standard beekeeping practices for population growth management and mite control. Half of the colonies were randomly assigned to the pollen deprived treatment, with a bottom-mount Sundance pollen trap (Ross Rounds, Canandaigua, NY, USA) installed to restrict the entry of pollen into the colonies. According to the manufacturer, these traps capture approximately 70% of pollen entering the hives. Traps were deployed every other day for a total of four pollen collection (deprivation) events. All the pollen collected from the apiary on each collection day was homogenized and redistributed as a pollen patty (mixed with a small amount of municipal tap water) to the pollen-supplemented treatment colonies. Pollen was redistributed to the supplemented colonies on each of the pollen collection days (*n* = 4), equaling a period of eight total days of supplementation or deprivation, encompassing a complete period of bee development from egg to pupa for a single cohort across the apiary, all simultaneously receiving their respective feeding treatment.

The pollen deprived and pollen supplemented treatments were replicated three times in 2017, once in March, May, and October. While the same colonies were used, pollen traps were removed and treatments re-randomized for each replicate. As the pollen collection events only spanned one week, there was sufficient time between replicates for colonies to recover given the egg laying potential of queens, particularly during the spring build-up period [24]. A summary of the amount of pollen collected per replicate is presented in Table 1. Field work was carried out in an agricultural area just south of Baton Rouge, LA primarily used for livestock, with little to no estimated use of neonicotinoids within the foraging region surrounding the apiary [25], or presence of neonicotinoid treated crops (corn and soybeans accounted for only 5.6% of total land use; https://nassgeodata.gmu.edu/CropScape/). Thus, we assumed little, if any, clothianidin was present in field-collected pollen.

### 2.2. Clothianidin and Laboratory Survival Assays

One week after the last deprivation/distribution event for each replicate, combs of mature pupae were transferred to emergence cages and adult bees emerged overnight in a humidified incubator, following Mogren, Margotta [16]. Bee cages (8 oz. Solo^®^ paper food cups, product no. VS508) and a clear plastic straw-slotted lid (Solo^®^ product no. 626TS) with 40 newly emerged bees were assigned to clothianidin treatments, with the colony serving as the unit of replication. Not all of the established 20 colonies were used in each replicate (March: pollen deprived *n* = 9, supplemented *n* = 9; May: pollen deprived *n* = 4, supplemented *n* = 6; October: pollen deprived *n* = 7, supplemented *n* = 7). For all replicates, colonies were only included if they were queen right and contained sufficient brood for downstream analyses. Unproductive colonies were excluded from a replicate to reduce potentially confounding effects associated with disease and high mite levels, though neither were monitored directly. Unseasonably heavy rains during May resulted in stalled brood production in some of the colonies, resulting in fewer colonies used for this replicate.

Clothianidin was administered in a 50% *v*:*v* solution of sucrose and municipal tap water at concentrations of 0, 10, 40, 200, and 400 µg/L, which reflect field realistic sublethal concentrations of clothianidin recovered in honey (10 µg/L) and pollen (40 µg/L) [5], the oral LD50 for adult honey bees (equivalent to 200 µg/L given average adult consumption of sucrose in the laboratory [16,26], and an acutely toxic concentration (400 µg/L). Each cage was provided with 1.5 mL of their respective clothianidin treatment in a microcentrifuge tube inserted through the straw hole. Feeders were replaced daily. Laboratory rearing practices were based on those recommended by [27], with the exception that caged bees did not receive a pollen supplement, as we hypothesized that this could confound the pollen deprived or supplemented colony-level treatments. Bee cages were maintained in a humidified incubator at 31 °C and mortality recorded daily for 10 days. Kaplan-Meier estimates were used to evaluate differences in adult bee survival between clothianidin concentrations and colony feeding treatments using a log-rank test. Survival data between replicates were pooled for analysis. Trends between sampling replicates (month) were not considered in order to maximize within-treatment sample size.

### 2.3. Stress Protein and Lipids Analysis

Sublethal effects associated with colony dietary stress were evaluated in late-instar larvae and adults. On the last day of pollen collection and redistribution in May and October, 10 fourth/fifth instar larvae were collected from frames. Larvae were not collected in March, as the initial intent of the study was to investigate nutritional deficit carry over into adulthood. However, the weather-mediated pollen dearth during colony build-up prompted us to follow up with a larval health component. Individual larvae were stored in 1.5 mL microcentrifuge tubes on ice until returning to the lab, where they were frozen at −80 °C until analysis. Newly emerged adults (as described above) were exposed to their respective clothianidin feeding treatments for three days prior to being frozen at −80 °C. Individual bee samples were analyzed for superoxide dismutase (SOD) to evaluate oxidative stress associated with dietary stress and pesticide exposure, and total lipids.

SOD activity was measured using a Superoxide Dismutase Assay Kit (Cayman Chemical, Ann Arbor, MI, USA, product no. 706002) [16]. Bees were homogenized in 500 µL of 50 mM buffer solution (50 mM sodium phosphate, 1 mM EDTA, 210 mM mannitol, 70 mM sucrose, pH 7.3) (whole body for larvae and abdomen only for adults) and centrifuged for 10 min at 4 °C at 10,000× *g*. Ten µL of the supernatant was diluted 1:500 with the supplied Sample Buffer, and the remainder of the assay conducted following the manufacturer’s instructions. SOD in unknowns was compared to a standard curve of SOD (provided by the manufacturer) and absorbance read at 450 nm using a SpectraMax 190 microplate reader (Molecular Devices, Sunnyvale, CA, USA). SOD activity was expressed as units of activity per min per mg protein (as measured using a Bradford protein assay with BSA standard).

Lipids were measured following Mogren and Lundgren [5] with a phosphovanillin assay. Briefly, lipids were separated from 25 µL of the homogenate using a methanol:chloroform extraction (300 µL, 2:1 methanol:chloroform). The supernatant was evaporated in a 10 × 75 mm glass tube on a heat block at 90 °C until approximately 25 µL of liquid remained, after which the sample was incubated for 2 min with 40 µL sulfuric acid at 90 °C. After cooling for 1 min on ice, 960 µL of vanillin-phosphoric acid reagent was added (600 mg vanillin dissolved in 100 mL ultrapure water, diluted to 500 mL with 85% phosphoric acid) and samples incubated for 25 min at 23 °C. Aliquots of 200 µL were transferred to a 96 well plate in duplicate and optical densities measured at 525 nm. Concentrations of lipids in unknowns were compared to a standard curve of 0, 1, 5, 10, 25, 50, 75, and 100 µg (54 µL olive oil in 50 mL chloroform).

Statistical differences between SOD activity and lipid concentrations in adults were, respectively, determined using two-way ANOVAs. The independent variables were colony-level feeding treatment (pollen deprived vs. supplemented) and clothianidin concentration. Because there was no significant difference between sampling replicate (March, May, October), this variable was excluded from models. Larval lipid data did not uphold assumptions of equal variance and thus were analyzed using a Wilcoxon Z-test, and SOD levels were analyzed using a two-sample *t*-test. Dependent variables for larvae were colony-level feeding treatment and sampling replicate (May, October). Colonies serve as the unit of replication, so differences between colonies were not considered.

## 3. Results

### 3.1. Clothianidin and Laboratory Survival Assays

**Figure 1:**
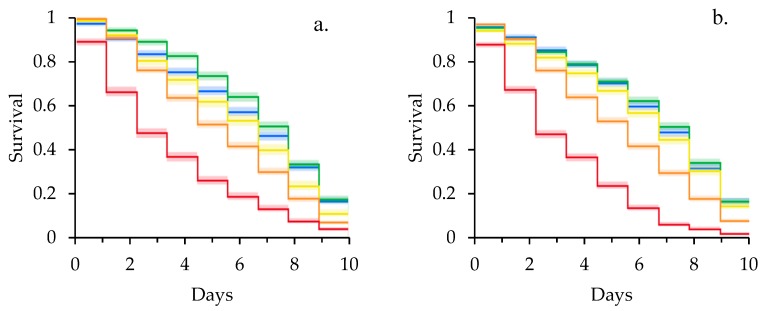
Kaplan-Meier survival curves for adult bees fed clothianidin reared in (**a**) pollen deprived and (**b**) pollen supplemented colonies. Shading represents 95% Confidence Intervals. Blue = 0 µg/L, Green = 10 µg/L, Yellow = 40 µg/L, Orange = 200 µg/L, Red = 400 µg/L.

Kaplan-Meier survival curves of newly emerged adult bees exposed to clothianidin revealed a significant effect of larval pollen deprivation on adult clothianidin susceptibility (Figure 1). Specifically, adult bees reared in pollen deprived colonies had higher rates of mortality in controls (χ^2^ = 7.73, df = 1, *p* = 0.005), at the sublethal concentration of 40 µg/L (χ^2^ = 28.4, df = 1, *p* < 0.001), and at 200 µg/L (χ^2^ = 8.65, df = 1, *p* = 0.003) than adult bees reared in pollen supplemented colonies. There was no significant difference in rates of mortality for bees fed 10 µg/L (χ^2^ = 1.53, df = 1, *p* = 0.216) or 400 µg/L (χ^2^ = 2.39, df = 1, *p* = 0.122) clothianidin when reared in pollen deprived and supplemented colonies. Within the pollen deprived feeding treatment (Figure 1a), mortality differed significantly between all concentrations of clothianidin (χ^2^ = 1412, df = 4, *p* < 0.001; 10 < 0 < 40 < 200 < 400 µg/L). In contrast, mortality at 0, 10, and 40 µg/L were statistically equivalent for pollen supplemented bees, which was in turn less than mortality at 200 and 400 µg/L (χ^2^ = 2637, df = 4, *p* < 0.001; 0 = 10 = 40 < 200 < 400 µg/L) (Figure 1b).

### 3.2. Stress Protein and Lipids Analysis

**Figure 2:**
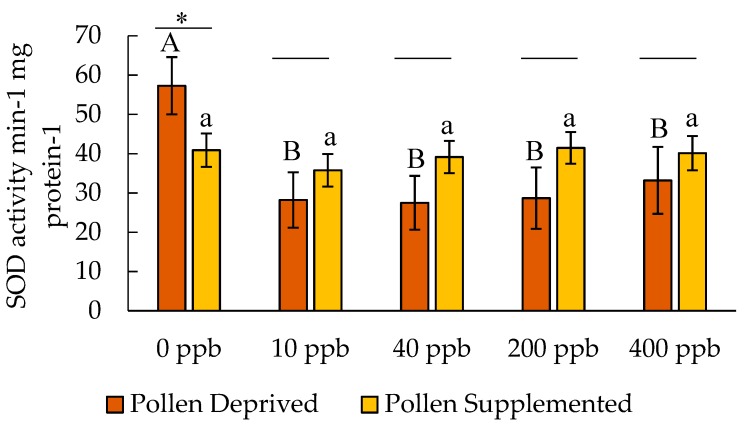
Activity of superoxide dismutase in adult honey bees after clothianidin exposure (mean ± SE). Letters indicate significant differences between doses within a feeding treatment, and * indicates a significant difference between feeding treatments for a particular dose.

SOD concentrations in adult honey bees were significantly affected by clothianidin dose (F = 3.01, df = 9,160, *p* = 0.019) (Figure 2). The dose by colony feeding treatment interaction was not significant (F = 2.26, df = 9,160, *p* = 0.065). There was no significant effect of larval feeding treatment on adult SOD levels (F = 1.56, df = 9,160, *p* = 0.213), though within the adult bees in the pollen deprived treatment, SOD was higher in controls than clothianidin treatments (F = 3.04, df = 4,667, *p* = 0.023).

**Figure 3:**
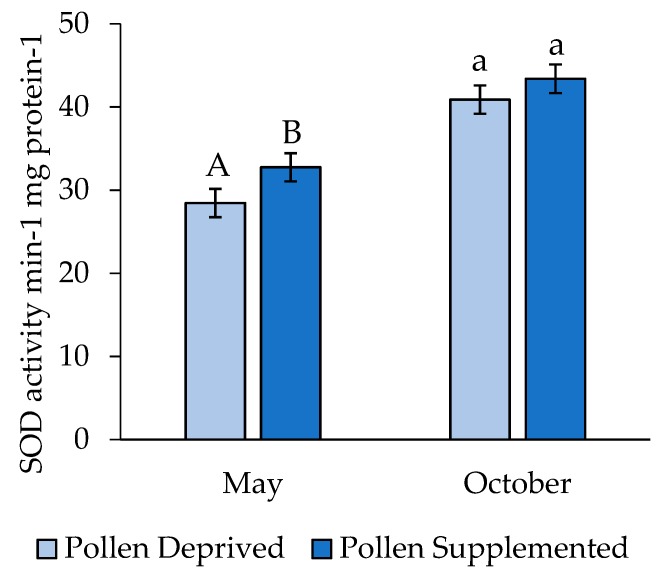
Activity of superoxide dismutase in 4th/5th instar honey bee larvae (mean ± SE). Letters indicate significant differences between feeding treatments within a replicate.

Larval SOD concentrations differed significantly by replicate (t = 4.99, df = 277, *p* < 0.001), and thus these replicates were analyzed separately. SOD levels were higher among larvae in the pollen supplemented treatment in May (t = 1.79, df = 119, *p* = 0.038), though there was no difference between larvae reared in pollen supplemented and deprived colonies in October (t = 1.04, df = 159, *p* = 0.152) (Figure 3).

**Figure 4:**
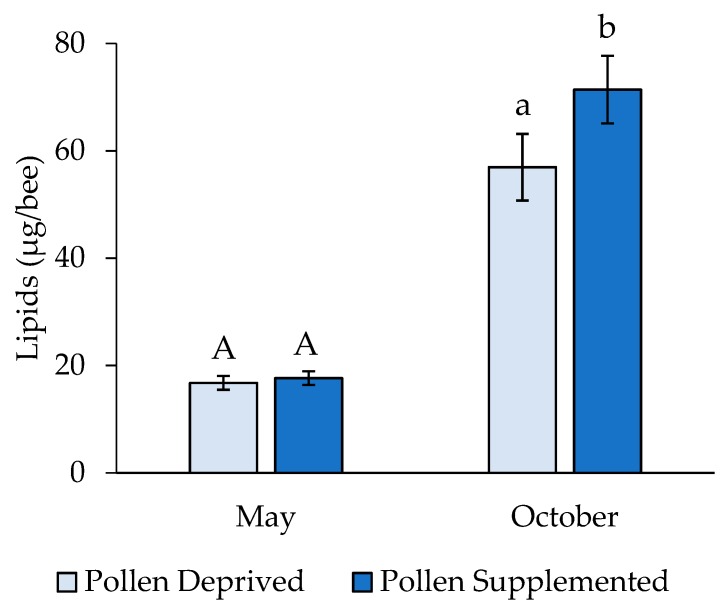
Lipid concentrations from 4th/5th instar honey bee larvae (mean ± SE). Letters indicate significant differences between feeding treatments within a replicate.

Larval lipid levels also differed significantly between seasonal replicates (Wilcoxon Z = −6.19, *p* < 0.001), and thus they were analyzed separately. In May, there was no significant difference in larval lipids between feeding treatments (t = 0.50, df = 119, *p* = 0.619), though larvae reared in pollen supplemented colonies had significantly greater lipid levels in October (t = 2.32, df = 159, *p* = 0.021) (Figure 4). Adult lipid levels were unaffected by larval feeding treatment and clothianidin dose (2-way ANOVA: F = 1.37, df = 9,176, *p* = 0.2035; pollen supplemented: 19.5 ± 2.38 µg/bee, pollen deprived: 23.1 ± 2.67 µg/bee).

## 4. Discussion

Our results indicate that nutritional stress for the duration of the larval period does carry through to the adult stage and can be measured as increased susceptibility to sublethal concentrations of clothianidin. When reared in pollen supplemented colonies, field-realistic concentrations of clothianidin (10–40 µg/L) did not affect survival of adults in cage experiments. However, when reared in pollen stressed colonies, mortality of adult bees was greater at 40 µg/L than controls, a concentration encountered in the pollen of untreated forage adjacent to seed-treated corn fields [5]. This indicates that nutritional stress may interact with pesticides as a secondary environmental stressor [28] and increase adult worker bee mortality. In the present study, colony level supplementation with 648 ± 159 g of pollen (calculated across the field season) was sufficient to help mitigate oral clothianidin as a secondary stressor. In our experimental set up, adults were also deprived of pollen during laboratory clothianidin exposures, where in the hive they would be consuming pollen. Thus, the amount of pollen required within a colony to mitigate stress may be less under more field realistic conditions.

When challenged simultaneously with nicotine (which affects insects similarly to neonicotinoids) and reduced brood rearing temperatures, Archer, CWW [15] found that the survival of African honey bees (*A. mellifera scutellata*) improved when adults were fed a high protein diet. In another study, survival was unaffected by oral imidacloprid exposure in honey bees fed pollen-supplemented diets, whereas mortality increased in those simultaneously exposed to dietary imidacloprid and protein-lacking diets [16]. Together, these results suggest that pollen as a dietary constituent is critical for mitigating stress associated with sublethal neonicotinoid exposures, which are found in bee-collected pollen worldwide [North America: Mogren and Lundgren [5], Lu, Chang [29], Codling, Al Naggar [30]; Europe: Botías, David [6], David, Botías [31], Sánchez-Hernández, Hernández-Domínguez [32], Tosi, Costa [33]; Africa: Codling, Al Naggar [34]].

When fed to larvae, neonicotinoid-contaminated pollen reduces both larval and pupal survival and increases stress protein activity [35]. Superoxide dismutase (SOD) is expressed during periods of oxidative stress [36], which can lead to premature aging [37], and background concentrations of this enzyme are likely maintained, even in healthy individuals. Above average activity levels would be indicative of oxidative stress, and an enhanced need for SOD. Li et al. (2014) found Cu/ZnSOD and MnSOD expression were significantly higher in newly emerged workers fed 35% protein supplements as larvae, so it is interesting that in the present study there was no significant difference in adult SOD activity between feeding treatments. Generally, adult SOD concentrations were similar to those reported in Mogren, Margotta [16]. However, adult bees reared in pollen deprived colonies had significantly greater SOD activity than paired bees reared in pollen supplemented colonies in the clothianidin control, indicating enhanced oxidative stress in the presence of nutritional stress. Further research is needed to better understand the mechanism governing this correlation and why it appears to moderate in simultaneous exposure to dietary neonicotinoids, particularly in the context of using SOD as a biomarker of sublethal stress.

To our knowledge, this is the first study to report SOD activities in honey bee larvae. Overall, these were comparable to concentrations in adult honey bees. Concentrations were lower in May than October, when heavy rains caused a pollen dearth across the apiary and surrounding landscape relative to amounts normally collected in previous years, though significant differences between feeding treatments were only seen in May, where SOD was higher in larvae from pollen supplemented colonies. This increase may indicate a rescue effect resulting from the necessary protein components being present to generate the enzymes for stress mitigation (Liao et al., 2017), though further research into the optimal concentrations of SOD in honey bees is needed. From our results, it would appear that SOD activity in larvae is a good indicator of sublethal stress associated with pollen dearth during development, and thus assays could be further developed to evaluate colony-level feeding stress for larvae.

Late instar larval lipid concentrations from May were less than one third of the lipid concentrations of larvae reared during a peak pollen flow (October). Additionally, a modest level of pollen supplementation in May did not result in significant differences between lipid concentrations in larvae, though larvae from supplemented colonies in October had higher lipid levels than the deprived treatment. In the larval stage, these lipids are important leading into pupation (Brodschneider and Crailsheim 2010). This lack of a treatment effect in May could be explained by larval lipid levels responding to pollen quality as opposed to pollen quantity [38], though this is not something we tested for directly. The complete nutritional requirements for honey bee larval development are not currently well understood [39], though the results of our study may help shed light on this, as 366 g of pollen per colony in May was not enough to boost overall larval lipid levels. As a bioindicator, larval lipid concentrations need further study in the context of larval nutrition.

Interestingly, adults from the May replicate did not perform significantly differently from adults of the March and October replicates with regards to survival after clothianidin feeding, indicating that if the larvae with low lipid concentrations survive to adulthood during widespread periods of dearth, there is not necessarily an additional survival disadvantage when exposed to a secondary pesticide stressor. However, we did not test this directly, and follow up is needed. Adult lipid levels were unaffected by feeding treatment, possibly as a result of the experimental design: no pollen was provided in the clothianidin exposure cages so as to eliminate any confounding effect on larval feeding treatments. However, with no protein source available the basic components for building and storing lipids would have been absent, and thus no effect was observed. Elsewhere, starvation at the larval stage has altered the physiology of energy storage in adults [40]. Dietary neonicotinoid exposures have also been shown to reduce honey bee adult lipid levels in the field [5] and thus lipid concentrations are a valid sublethal marker in adult honey bees, though the extent to which this is measurable in laboratory experiments in the context of pollen deprivation requires further optimization.

## 5. Conclusions 

Pollen quantity has previously been shown to promote colony strength in agricultural areas [41]. Additionally, we demonstrate that colony-level pollen deprivation results in measurable physiological differences in larval and subsequent adult stages. In areas where heavy use of neonicotinoids may result in honey bee exposure, it is critical that abundant pesticide-free pollen resources be available for brood rearing and subsequent stress mitigation. Beekeepers should consider storing or purchasing field-collected pollen such that during periods of pollen dearth, colonies may be supplied with locally-collected pollen to mitigate oxidative stress of larvae within colonies, and improve adult worker longevity should they encounter dietary pesticides.

## Figures and Tables

**Table 1:** Total pollen amounts collected and distributed for each replicate.

Replicate	Total Pollen (kg) [Range] ^1^	N ^2^	Distributed (g) ^3^
March	5.97 [0.77–3.07]	9	664
May	2.19 [0.45–0.72]	6	366
October	6.41 [1.05–2.28]	7	915

^1^ The range of pollen collected on collection days (*n* = 4) within the apiary per replicate. ^2^ Refers to the number of pollen supplemented colonies per replicate. ^3^ The total amount of pollen distributed to the pollen supplemented treatment colonies.
